# Adiponectin’s Role in the Development and Progression of Differentiated Thyroid Carcinoma: A Narrative Review of Current Evidence

**DOI:** 10.7759/cureus.109107

**Published:** 2026-05-18

**Authors:** Ruxandra Daniealopol, Valentin Daniealopol, Tatiana Daniela Sala, Radu Neagoe

**Affiliations:** 1 Department of Surgery, George Emil Palade University of Medicine, Pharmacy, Science, and Technology of Targu Mures, Targu Mures, ROU; 2 2nd Department of Surgery, George Emil Palade University of Medicine, Pharmacy, Science, and Technology of Targu Mures, Targu Mures, ROU

**Keywords:** adipokines, adiponectin, differentiated thyroid carcinoma, obesity, thyroid cancer

## Abstract

The incidence of differentiated thyroid carcinoma has risen dramatically worldwide in recent years, paralleling the increasing obesity rates. There is compelling evidence that identifies obesity as an independent risk factor for thyroid cancer, while highlighting the role of low-grade chronic inflammation, metabolic changes, and abnormal adipokine secretion. Adiponectin (APN) is an abundant adipokine in human serum that particularly stands out due to its inverse correlation with adipose tissue abundance and its well-established characteristics: anti-inflammatory, anti-proliferative, pro-apoptotic, and insulin-sensitizing properties. This review aims to synthesize the current state of evidence and research on the association between APN and thyroid carcinoma. It focuses on epidemiological links, including gender-specific effects and correlations with tumor aggressiveness features. The paper explores molecular mechanisms while aiming to underscore important signaling pathways such as AMP-activated protein kinase and the APN receptor-2-UNC-51-like kinase-1 autophagy axis. Moreover, it seeks to evaluate the potential of APN as a biomarker for both diagnosis and risk stratification, with particular focus on receptor expression and adipokine ratios. A deeper understanding of the APN’s role might reveal new directions for primary prevention, timely diagnosis, and personalized treatment of thyroid neoplasms.

## Introduction and background

Differentiated thyroid neoplasms, namely papillary thyroid carcinoma (PTC) and follicular thyroid carcinoma (FTC), account for more than 95% of all thyroid cancers (TCs) [1,2]. Recently, the global prevalence and incidence of thyroid neoplasms have witnessed a substantial increase, making it the most common type of endocrine cancer in Europe and worldwide [3,4]. Even though some might try to attribute this solely to the growing use of diagnostic tools, such as ultrasonography and fine-needle aspiration biopsy (FNAB), it is hard to believe the numbers are entirely due to increased detection; hence, the need for further exploration. Both researchers and clinicians have observed that there is a consistent association linking the rising incidence of thyroid malignancies with the concomitant global obesity pandemic. Growing evidence identifies obesity as an independent risk factor for differentiated thyroid carcinoma (DTC) development and progression, thus turning obesity management into a vital public health strategy for primary prophylaxis [5].

Even though the definition of obesity may slightly vary from one country to another, the WHO defines it as a chronic condition with a BMI higher than 30 kg/m^2^, and typically associates, besides low-grade inflammation, a series of comorbidities that include: arterial hypertension, dyslipidemia, type 2 diabetes, sleep apnea, etc. The WHO reports that on a global scale, obesity has tripled since 1975; also, in 2016, almost two billion adults were overweight, and 13% of the world population (18 years old and above) were obese [6]. Although BMI is not the most accurate indicator, it is recommended for measuring body fat, along with a general indication for maintaining values between 18.5 kg/m^2^ and 24.9 kg/m^2^. Obesity is characterized by numerous metabolic abnormalities, including low-grade chronic inflammation and modified insulin resistance, which are believed to significantly increase the risk of various cancer types [7-9]. In overweight and obese patients, adipose tissue dysfunction invariably leads to dysregulation in the secretion of a wide range of molecules called adipokines or adipocytokines [10,11].

Adiponectin (APN) is a key anti-inflammatory cytokine secreted by adipose tissue, accounting for approximately 0.05% of total plasma protein, that has been proven to be inversely associated with adiposity [12]. Considering this inverse relationship along with APN’s antiproliferative, proapoptotic, antiatherogenic, and insulin-sensitizing properties, we can establish APN as an endogenous protective factor. This suggests that the reduced levels in overweight and obese individuals exacerbate insulin resistance and chronic inflammation, hence promoting tumorigenesis [13-15]. Despite growing interest in APNs’ role in TC, the precise underlying mechanisms involved in DTC pathogenesis remain incompletely understood. This review aims to synthesize the current state of research on APN and DTC, focusing on their biological functions, possible associations with TC risk and progression, and potential as biomarkers.

## Review

The literature search for this narrative review followed a rigorous methodology to ensure comprehensive coverage of the existing publications. An attentive search was performed on the PubMed, SCOPUS, Web of Science, and Cochrane Library databases using the following keywords: "Adiponectin" OR "AdipoR1" OR "AdipoR2" AND "Thyroid cancer" OR "Thyroid carcinoma" OR "Thyroid neoplasm" covering studies published up to 2025, to ensure the inclusion of the latest published articles. Original research and review papers were taken into consideration.

The results were filtered based on several inclusion criteria: publications in peer-reviewed journals, human studies, papers written in English, and papers focusing on the role of APN in TC. The exclusion criteria include: duplicate articles, case reports, studies with no full text available, and studies written in languages other than English. Additionally, a manual search of the reference list was performed in order to identify other relevant articles. We screened titles, abstracts, and full-text publications and selected only those research papers that fit the mentioned criteria. Both quality assessment and data extraction were performed by two distinct reviewers, and any discrepancies were subsequently reviewed and resolved by a third senior researcher.

Considering this paper was designed as a narrative review aiming to synthesize evidence from heterogeneous sources, including observational studies, case-control studies, meta-analyses, and reviews, a formal risk-of-bias or evidence-grading framework was not applied. Nevertheless, there are several limitations to this paper as most studies have small sample sizes, observational designs, and some generate inconsistent or contradictory findings that can be attributed, to a certain extent, to potential confounding factors such as BMI, the presence of metabolic syndrome or diabetes mellitus, differences in assay methods, or demographic variables.

Adipocyte dysfunction and the adiponcosis hypothesis

Lately, excess weight has been consistently associated with tumorigenesis, as well as tumoral progression in different forms of cancer. Numerous researchers have published data regarding the so-called "obesity-related cancers," but only a few managed to identify and explain the exact mechanisms involved, as these are highly complex and most likely distinct for each particular type [7,9,15].

"Adiponcosis" is a concept used to describe the direct involvement of dysfunctional adipose tissue in the development and progression of different types of cancer. While in a healthy physiological state, APN serves as a protective factor by maintaining metabolic homeostasis and inhibiting systemic inflammation. In obesity, the hypertrophy of adipose tissue leads to a shift in adipocytes’ secretory profile [16]. Increased stress and hypoxia at the level of hypertrophic adipocytes initiate the recruitment of macrophages and other immune cells within the adipose tissue. These cells will further secrete pro-inflammatory molecules, including tumor necrosis factor-alpha (TNF-α) and interleukin-6 (IL-6), which suppress APN production. Low levels of APN are associated with systemic insulin resistance and consequent hyperinsulinemia. High insulin levels trigger the production of insulin-like growth factor 1 (IGF-1), meanwhile decreasing its binding protein levels, insulin-like growth factor binding protein 1 (IGFBP-1) and insulin-like growth factor binding protein 2 (IGFBP-2). This results in increased levels of bioavailable IGF-1, which will activate the phosphatidylinositol 3-kinase/protein kinase B (PI3K/Akt) and mitogen-activated protein kinase (MAPK) pathways in thyroid follicular cells, therefore promoting uncontrolled proliferation and inhibiting apoptosis [16-18]. APN’s ability to reverse these metabolic alterations suggests its potential as a natural brake on thyroid tumorigenesis, a brake that is malfunctioning in the context of excess adiposity and obesity.

APN: biology and implications in carcinogenesis

To properly understand the local and systemic impact of APN on thyroid carcinoma, one must first become familiar with the unique biochemical structure and its receptor dynamics. APN is made of 244 amino acids, its structure including a variable region, a collagen-like domain, an N-terminal signal peptide, and a C-terminal globular domain. APN presents three distinct isoforms: low-molecular-weight trimers, middle-molecular-weight hexamers, and high-molecular-weight multimers. These isoforms possess different tissue specificity and different affinity for their specific receptors, contributing to the varied biological actions of APN [19].

APN mediates its effects through binding to three distinct receptors: adiponectin receptor 1 (AdipoR1), adiponectin receptor 2 (AdipoR2), and T-cadherin. The first is ubiquitously expressed, with particularly high concentrations at the level of the endothelium and skeletal muscles, while the second can be found predominantly in the liver and white adipose tissue. T-cadherin is a receptor similar to the cadherin family, which is selectively expressed in intratumoral capillary vessels and is believed to play a part in mediating certain effects, one of the most important being angiogenesis [20,21]. The distinctive membrane configuration of AdipoR1 and AdipoR2 differentiates them from other G-protein-coupled receptors through the presence of an internal N-terminus and an external C-terminus [22]. Consistently, numerous cancer cell lines (including those derived from thyroid neoplasms) have been shown to express AdipoR1 and AdipoR2 [23-25].

The diverse effects of APN on inflammatory, metabolic, and cellular pathways highlight its role as a crucial mediator; it actively opposes numerous obesity-driven carcinogenic pathways, one of the most representative being the insulin/IGF-1 axis [26,27]. Conversely, decreased APN levels reduce the protective effect against tumorigenesis, thereby positioning it as a suitable candidate for novel targeted therapies aimed at disrupting the obesity-cancer link.

The mechanisms linking obesity, APN, and cancer are indisputably multifaceted and complex. Increased adiposity, along with reduced APN levels, will gradually lead to insulin resistance and compensatory hyperinsulinemia. High insulin levels sequentially lead to decreased synthesis of IGFBP-1 and IGFBP-2 at the level of the liver, thus generating increased levels of bioavailable IGF-1. IGF-1 and insulin are known to promote cellular proliferation and inhibit apoptosis by signaling through their specific receptors (insulin receptor and IGF-1 receptor) and by stimulating vascular endothelial growth factor synthesis, hence contributing to tumorigenesis [28,29].

APN is a powerful insulin sensitizer, and this is particularly relevant considering that hyperinsulinemia and insulin resistance are already established risk factors for cancer development [30,31]. Additionally, APN possesses strong anti-inflammatory effects, counteracting obesity-related low-grade chronic inflammation; it specifically inhibits pro-inflammatory cytokines such as TNF-α and IL-6, while at the same time, it stimulates the secretion of anti-inflammatory cytokine IL-10. Even though it is well established that APN is responsible for inducing apoptosis and limiting cell proliferation, its complex role in angiogenesis still requires further investigation; so far, both pro- and anti-angiogenic effects have been reported, depending on the particular context or experimental conditions [26,27,30]. This contradiction could suggest that the overall anti-tumor effect derives from the dominance of its pro-apoptotic and anti-proliferative properties over any pro-angiogenetic effect within the tumor microenvironment. Undoubtedly, defining the specific molecular contexts or profiles that determine whether APN exerts a pro- or anti-angiogenic effect remains an area of interest for further research.

The association between APN, thyroid carcinoma risk, and tumor aggressiveness

A study conducted by Mitsiades and his collaborators as early as 2011 showed that patients with TC presented significantly lower APN levels than the control group (17.00±6.32 µg/mL vs. 19.26±6.28 µg/mL; p<0.001). Moreover, patients in the highest tertile of circulating APN concentrations had significantly lower chances of developing any type of thyroid neoplasm (OR=0.29; 95% CI: 0.16-0.55) or PTC (OR=0.27; 95% CI: 0.14-0.55) [24]. Regarding the link between APN levels and the aggressive clinicopathological features of DTC, cancer patients who were obese exhibited significantly lower serum levels than lean patients, the difference being particularly pronounced in females [5]. Additionally, serum APN was negatively correlated with several aggressive features of PTC, such as extrathyroidal extension (ETE), lymph node metastasis, and tumor diameter over 10 mm, indicating that decreased APN levels are associated with more aggressive behavior of PTC in patients with obesity [32]. Another publication reinforces this link by revealing a significant association between increased BMI and aggressive characteristics of PTC, establishing high BMI as an independent risk factor for lymph node metastasis and ETE [33]. This confirms the hypothesis that lower APN, as a consequence of higher BMI/adiposity, contributes to tumor progression and a more aggressive disease.

The European Prospective Investigation into Cancer and Nutrition (EPIC) study, a large-scale prospective study, showed an inverse association between circulating APN levels and the risk of developing TC. However, the association was not demonstrated in men (OR for the third vs. first tertile = 1.39, 95% CI: 0.67-2.76, p-trend = 0.37); it is only specific for women (OR for the third vs. first tertile = 0.69, 95% CI: 0.49-0.98, p-trend = 0.04), suggesting a gender-specific protective effect [5]. This gender-specific aspect, in particular, the inverse association observed in female but not male patients, is highly important for multiple reasons. Considering the higher incidence of thyroid carcinoma among women and the already established effect of obesity on endogenous sex hormones in increasing the cancer risk, this suggests APN, adiposity, and sex steroids are all part of an intricate mechanism leading to thyroid carcinogenesis. APN’s protective effect against DTC seems to be more obvious if not completely restricted to women, indicating that the anti-tumor effects might be modulated through or interact with gonadocorticoid pathways [5,33,34]. Further research is necessary to clarify the exact mechanisms of APN-sex hormone crosstalk within the endocrine system in general and the thyroid cell in particular. This also suggests that future prophylactic or therapeutic strategies targeting APN should consider a gender-based personalized approach.

Though the majority of published research supports an inverse correlation, the literature shows some heterogeneity (Table 1). For example, a study conducted by Kwon et al. [35] reported no statistically significant difference in median APN levels between the PTC group and the benign nodule group (5.4 µg/mL vs. 6.3 µg/mL). In a similar manner, Abooshahab et al. showed that there is no significant change in serum APN levels in the medullary carcinoma group compared with controls, hence suggesting that the metabolic link is solely specific to follicular origin tumors [36].

**Table 1:** Studies investigating APN levels in patients with DTC APN, adiponectin; IGF1, insulin-like growth factor 1; OR, odds ratio; PTC, papillary thyroid carcinoma; TC, thyroid cancer.

Author	Year	Title	Study type	Sample (TC/control)	Main findings
Mitsiades et al. [24]	2011	Circulating adiponectin is inversely associated with risk of thyroid cancer: in vivo and in vitro studies	Case-control	175/107	APN levels significantly lower in TC patients; OR=0.29 for high APN tertile
Dossus et al. [5]	2018	Adipokines and inflammation markers and risk of differentiated thyroid carcinoma: the EPIC study	Case-control	475/1016	APN levels inversely correlated with TC risk in women
Mele et al. [37]	2019	Circulating adipokines and metabolic setting in differentiated thyroid cancer	Case-control	30/47	APN levels significantly lower in TC than benign nodules and healthy controls
Warakomski et al. [39]	2018	Concentrations of selected adipokines, interleukin-6, and vitamin D in patients with papillary thyroid carcinoma in respect to thyroid cancer stages	Clinical-observational	177	Low APN levels in PTC patients with metabolic syndrome compared with those without
Kwon et al. [35]	2020	Serum adiponectin and progranulin level in patients with benign thyroid nodule or papillary thyroid cancer	Case-control	131/26	Median APN values lower in PTC than benign nodules (5.4 vs. 6.3, p=0.6)
Pazaitou-Panayiotou et al. [31]	2016	Serum adiponectin and insulin-like growth factor 1 in predominantly female patients with thyroid cancer: association with the histologic characteristics of the tumor	Clinical- observational	175	IGF1/APN ratio independently associated with tumor dimensions

The discrepancies can be partially, but not entirely, explained by differences in sample size, ethnic background, or the inclusion of metabolic syndrome as a variable [37,38]. For instance, Warakomski et al. showed that even though serum APN might not correlate with tumor size for the entire PTC population, it is decreased significantly in PTC individuals with metabolic syndrome. This might imply that APN acts as a critical component of the metabolic setting predisposing some patients to a more aggressive disease [39]. 

APN receptors expression and pathological correlations

Beyond their systemic levels, AdipoR1 and AdipoR2 expression within thyroid tissue offers valuable clues regarding the clinical behavior of thyroid carcinoma. The receptors act as mediators of APN’s tumor-suppressive signals; therefore, their dysregulation often signals the presence of advanced disease [32,34]. Even though AdipoR1 and AdipoR2 have been shown to be present in both PTC tissue and normal paratumor thyroid tissue, the intensity and homogeneity of their expression can vary significantly. Cheng et al. conducted research evaluating the expression of APN receptors within 82 TC tissues using immunohistochemistry and showed that AdipoR1 was expressed in 27% of tumors, while AdipoR2 was present in 47% [32]. Moreover, the absence of these receptors was significantly associated with more aggressive tumor features, such as ETE, higher TNM staging, and multicentricity. Furthermore, individuals with AdipoR1 and AdipoR2 expression showed a trend toward decreased cancer-free survival [32,33]. This suggests that the loss of the APN signaling axis might play a critical role in the progression of DTC.

APN receptor downregulation in TC is not solely a passive result of cellular transformation but can be driven by active molecular interactions. Studies have shown that histone deacetylase inhibitors, such as valproic acid and trichostatin A, can modify AdipoR1 and AdipoR2 expression in TC cell lines [32,40,41]. This indicates that epigenetic modifications may play a part in inhibiting APN's protective role during tumor progression. In addition, the low-grade chronic inflammation associated with obesity, defined by increased TNF-α levels, can lead to a compensatory downregulation of APN receptors, further aggravating APN resistance observed in patients with obesity (Table 2) [42,43].

**Table 2:** Studies focusing on APN receptors in DTC AdipoR, adiponectin receptor; AdipoR2-ULK1, adiponectin receptor-2-UNC-51-like kinase-1; FTC, follicular thyroid carcinoma; mRNA, messenger ribonucleic acid; PTC, papillary thyroid carcinoma.

Author	Year	Title	Model	Main findings
Cheng et al. [32]	2013	Expression and biologic significance of adiponectin receptors in papillary thyroid carcinoma	Human tissue and cell lines	AdipoR absence linked to higher TNM stage and multicentricity
Mitsiades et al. [24]	2011	Circulating adiponectin is inversely associated with risk of thyroid cancer: in vivo and in vitro studies	Human tissue and cell lines	AdipoR1/R2 mRNA detected in 32 PTC and 6 FTC/Hurtle specimens
Li et al. [40]	2024	Adiponectin inhibits the progression of obesity-associated papillary thyroid carcinoma through autophagy	PTC cell lines	AdipoR2-ULK1 pathway identified as mediator of antitumor effects

Molecular pathogenesis

Obesity induces a prolonged systemic inflammatory state known as low-grade chronic inflammation. The mechanisms through which APN counteracts this state are complex and involve the interaction of multiple intracellular signaling cascades (Table 3). The most prominent of these are probably the AMPK, mammalian target of rapamycin (mTOR), and UNC-51-like kinase-1 (ULK1) pathways.

**Table 3:** Molecular pathways involved in APN-DTC relationship AdipoR2/ULK1, adiponectin receptor-2-UNC-51-like kinase-1; AMPK/mTOR, AMP-activated protein kinase/mammalian target of rapamycin; APN, adiponectin; DTC, differentiated thyroid carcinoma; EMT, epithelial-mesenchymal transition; IKK, IkB kinase; MAPK/ERK, mitogen-activated protein kinase/extracellular signal-regulated kinase; PI3K/Akt, phosphatidylinositol 3-kinase/protein kinase B; TC, thyroid cancer; TNF-α/NF-kB, tumor necrosis factor-alpha/nuclear factor-kappa B.

Pathway/axis	Mechanism	Impact on TC cells	Key studies
AMPK/mTOR	Activation of p-AMPK, inhibition of p-mTOR and p-p70S6K	Reduced synthesis of proteins, cell cycle arrest	[44-46]
AdipoR2/ULK1	Phosphorylation of ULK1 at Ser555	Cellular differentiation and induction of autophagy	[40,47,48]
PI3K/Akt	p-Akt suppression through insulin sensitization	Inhibition of cell migration and survival	[40,44]
TNF-α/NF-kB	Inhibition of IKK activity	Reduced cytokine production, anti-inflammatory effect	[32]
MAPK/ERK	Attenuation of extracellular signal	Decreased epithelial-mesenchymal transition and invasive	[49]
E-cadherin/Snail	EMT markers modulation	Reduced migration and metastatic potential	[49]

AMPK is the primary intracellular sensor of energy status and the main mediator of APN effects. Its importance also arises from its capacity to disrupt both cellular behavior and metabolic regulators. In addition, AMPK activation induces the expression of p53 and p21 tumor suppressor proteins, which are known for their essential role as regulators of cell cycle arrest. When binding to AdipoR1 and AdipoR2, APN triggers the phosphorylation of AMPK; once activated, the latter serves as a metabolic checkpoint that antagonizes the mTOR pathway [44,45]. The mTOR pathway plays an essential role in thyroid tumorigenesis by promoting protein synthesis and cell cycle progression. Studies using TC cell lines have previously shown that APN and its agonists inhibit mTOR phosphorylation and its downstream effector, p70S6 kinase [46]. Therefore, by curbing this axis, APN limits both energetic and biosynthetic resources available for the rapid proliferation of tumoral cells.

An important breakthrough in understanding APNs’ role in DTC came along with the identification of the autophagy pathway as a mediator for its anti-tumor effects. Autophagy is a complex process consisting of cellular degradation and recycling that often acts like a tumor-suppressing mechanism by ensuring cellular quality control. Recent research looked into a novel AdipoR2-ULK1-p-ULK1Ser555 axis in PTC and demonstrated not only that APN stimulates the phosphorylation of ULK1 at the Ser555residue but also that activation of this axis induces increased lysosomal activity and autophagy [40]. The induction of autophagy directly inhibits TC progression and metastasis while promoting the differentiation of cancer cells, hence reducing their aggressive behavior [47,48]. Moreover, knockdown of AdipoR2 or ULK1 was proven to significantly diminish the pro-autophagic and anti-proliferative effects of APN agonists, thus confirming pathway specificity for thyroid carcinoma cells [40,48].

The aggressive features of some DTCs, particularly those associated with the BRAF V600E mutation, are characterized by the induction of epithelial-mesenchymal transition (EMT). Throughout EMT, tumor cells lose their epithelial characteristics (such as E-cadherin expression) while gaining mesenchymal features (for example, N-cadherin and Snail expression), facilitating the invasion of surrounding tissue and metastasis. APN has been demonstrated to modulate the expression of these EMT-related markers, probably by inhibiting the mTOR and MAPK pathways [49]. It is to be noted that this anti-metastatic property is particularly relevant for patients with obesity, who tend to present with more aggressive DTC forms, including higher rates of ETE and lymph node metastases.

Therapeutic potential of APN agonists

The evident anti-tumorigenic properties of APN have led to increased interest in its therapeutic potential [50]. Even so, the use of the full-length protein in clinical practice is challenging due to several reasons: low stability, complex oligomerization, and high plasma levels. This has prompted the need for developing synthetic APN receptor agonists.

AdipoRon falls precisely into this category. It is a small-molecule agonist, capable of binding to both AdipoR1 and AdipoR2; unlike the full-length protein, AdipoRon is orally active, has a long half-life, and is easily absorbed [51]. The latest research has shown that AdipoRon is able to inhibit both proliferation and migration of PTC cell lines and limit the energy metabolism in TC cells by suppressing glycolysis-related genes [18]. In addition, it can induce apoptosis and autophagy mechanisms through the AdipoR2-ULK1 axis.

Due to its ability to mimic the protective effects of APN without the physiological hurdles that come along with protein therapy, it is now being investigated as an adjuvant therapy for obesity-related thyroid neoplasms [51,52]. However, no large clinical trials have yet validated the use of APN agonists in TC, but promising results in other obesity-related models (such as breast and pancreatic cancer) provide a strong rationale for their clinical translation to patients with TC [51,53].

Present context and future implications

In this literature review, by synthesizing clinical, pathological, and molecular data, we aim to highlight the robust, multifaceted role of APN in TC. APN acts as a pivotal metabolic regulator and link between adipose tissue and the thyroid follicular cells.

APN’s role in thyroid carcinoma can be briefly summarized as a dual protective system, characterized, on the one hand, by systemic metabolic control and, on the other, by its function as a local signaling regulator [17]. Existing epidemiological data underscore that there may be a more pronounced protective effect of APN in women than in men, thereby suggesting that the management of obesity-related TC risk within female populations should particularly focus on metabolic health and bringing APN levels back to normal values [5]. In addition, age seems to be a significant factor influencing the relationship between adiposity and the risk of developing thyroid neoplasms; some publications suggest that increased BMI is a more potent risk factor in young and middle-aged individuals than in the elderly [33,34].

The local expression of APN receptors might offer a promising new tool for clinicians. In TC cases in which FNAB or histopathological examination of surgical specimens shows complete loss of AdipoR1 and AdipoR2 receptors, a more aggressive surveillance and treatment strategy should be considered, as these tumors are more likely to exhibit multifocality and ETE [24,32].

An essential question future research needs to address is how circulating APN levels correlate with the expression of APN receptors within the tumor microenvironment, and whether persistent low circulating APN levels lead to compensatory upregulation of its receptors within thyroid tumor cells. This reiterates the need for integrated studies that concomitantly evaluate systemic and tissue-specific APN parameters.

## Conclusions

The role of APN in the development and progression of DTC has evolved over the past years, from what was initially a simple correlation with excess weight and adiposity to what is now a complex signaling axis, with significant implications in terms of diagnostic, clinical, and therapeutic implications. The evidence gathered for this review validates that hypoadiponectinemia is truly a hallmark of the pro-tumorigenic metabolic status associated with obesity and TC. Furthermore, the inverse correlation between circulating APN and cancer risk, combined with the prognostic value of APN receptors, positions this as a central research theme in modern endocrine oncology.

The possible cause for which the subject stimulates such interest among the medical world is the fact that overweight/obesity is a modifiable risk factor for cancer, and the possible implications directly or indirectly derive from this. Future research should clarify whether the efficacy of synthetic APN agonists translates into long-term human trials and investigate APN's potential as a biomarker for screening high-risk obese populations. Moreover, cross-talk between APN and other molecules, such as obestatin and progranulin, warrants further exploration to build an accurate model of the adipocytokine network in thyroid tumorigenesis. As the medical community continues to confront the concomitant challenges of rising obesity and TC incidence, the APN signaling axis seems to be offering a vital target for both prevention and targeted therapy.
